# Molecular Basis of Anxiety: A Comprehensive Review of 2014–2024 Clinical and Preclinical Studies

**DOI:** 10.3390/ijms26115417

**Published:** 2025-06-05

**Authors:** Ermis Merkouris, Alexandra Brasinika, Meropi Patsiavoura, Chrysanthi Siniosoglou, Dimitrios Tsiptsios, Andreas S. Triantafyllis, Christoph Mueller, Ioulia Mpikou, Myrto T. Samara, Nikolaos Christodoulou, Konstantinos Tsamakis

**Affiliations:** 1 Neurology Department, Democritus University of Thrace, 68100 Alexandroupoli, Greece; ermimerk@med.duth.gr (E.M.); dtsipt@auth.gr (D.T.); 2 3rd Neurology Department, Aristotle University, 54124 Thessaloniki, Greece; aabrasinika@gmail.com (A.B.); meropepatsiaboura@gmail.com (M.P.); siniosoglouanthi@gmail.com (C.S.); 3 Department of Cardiology, Asklepeion General Hospital, 16673 Athens, Greece; andreas.triantafyllis@gmail.com; 4 Institute of Psychiatry, Psychology and Neuroscience (IoPPN), King’s College London, London SE5 8AB, UK; christoph.mueller@kcl.ac.uk; 5 Biomedical Research Center, South London and Maudsley NHS Foundation Trust, London SE5 8AF, UK; 6 Department of Molecular Biology and Genetics, Democritus University of Thrace, Dragana, 68100 Alexandroupolis, Greece; julia.v.mpikou@gmail.com; 7 Department of Psychiatry, Faculty of Medicine, University of Thessaly, 41110 Larisa, Greece; mysamara@uth.gr (M.T.S.); nichrist@uth.gr (N.C.); 8 Institute of Medical and Biomedical Education, St George’s, University of London, London SW17 0RE, UK; 9 Department of Clinical Sciences, New Anglia University, George Hill AI-2640, Anguilla

**Keywords:** anxiety, molecular, mechanisms, neurobiological, genetic, stress, treatment

## Abstract

Anxiety disorders are among the most common psychiatric conditions that significantly impair one’s quality of life and place a significant burden on healthcare systems. Conventional treatments have certain restraints, such as potential side effects and limited efficacy. Τhe underlying pathophysiological mechanisms of anxiety are not fully understood. A comprehensive literature search was performed in MEDLINE and Scopus databases for original English-language articles published between January 2014 and December 2024. Study selection, data extraction, and screening were independently carried out by multiple investigators using predefined criteria. Our review aimed to help better comprehend the molecular basis of anxiety, focusing on the hypothalamic–pituitary–adrenal (HPA) axis, serotonergic signaling, and gamma-aminobutyric acid (GABA) neurotransmission. In addition, we addressed the role of epigenetics and pharmacogenomics in personalized treatment. Although novel anxiety treatments are promising, they are predominantly preclinical and highly heterogeneous, which poses a challenge to achieving reliable therapeutic efficacy. Our findings could potentially contribute to the development of new therapeutic interventions. Further research is warranted, especially in human subjects, with an aim to combine genetic and epigenetic profiles to refine treatment approaches and develop innovative therapeutics.

## 1. Introduction

The stress of modern living significantly affects daily functioning and overall mental well-being [1]. Chronic stress is a significant risk factor in the emergence of various psychiatric conditions, such as generalized anxiety disorder (GAD), panic disorders, social anxiety disorder (SAD), post-traumatic stress disorder (PTSD), and specific phobias [2,3,4]. Based on findings from large-scale population surveys, approximately one third of individuals will experience an anxiety disorder at some point in their lives. Anxiety disorders display distinct patterns of onset and prevalence [5,6].

The pathophysiology behind anxiety is rooted in the interplay of neurobiological, genetic, and environmental factors. Stressful experiences during early development may lead to the sustained activation of the HPA axis, which can extend into adulthood; this sustained activation may increase vulnerability to anxiety disorders, emphasizing the long-term impact of early developmental stress on mental health [7,8]. In addition, the role of neurotransmitters in facilitating communication between brain regions is critical in understanding anxiety disorders. Enhanced activity in emotion-processing regions, observed in individuals with anxiety disorders, may stem from diminished inhibitory signaling mediated by γ-aminobutyric acid (GABA). Furthermore, recent studies have identified the involvement of serotonin (5-hydroxytryptamine, 5-HT), norepinephrine (NE), and dopamine (DA) in the pathogenesis of mood and anxiety disorders, highlighting their contribution to the neurobiological mechanisms underlying these conditions [9]. In the central nervous system, classic neurotransmitters are often co-released with neuropeptides, which are abundantly expressed in specific regions, where they influence circuits related to stress and emotion. Neuropeptides strongly associated with psychopathology include cholecystokinin (CCK), as well as galanin and neuropeptide Y (NPY), whilst vasopressin (AVP) and oxytocin are not only involved in the physiology of stress, but may also be clinically important for new treatment strategies [9,10,11]. Moreover, in recent years, the gut microbiome has been shown to play a pivotal role in the underlying pathophysiology of several neuropsychiatric disorders, including anxiety, by modulating the bidirectional communication between the gut and brain, collectively forming the microbiome–gut–brain (MGB) axis [12,13,14].

In recent decades, several genetic variants predisposing to anxiety disorders have been identified in different genome-wide association studies (GWASs), with variants within TMEM132D being among the earliest genetic associations identified in relation to panic disorder and anxiety severity [15]. However, the candidate genes are largely similar across different diagnoses and tend to be genes, products of which regulate the HPA axis and monoaminergic signaling [9]. The intronic region of NTRK2, which encodes the tropomyosin receptor kinase B (TrkB) for brain-derived neurotrophic factor (BDNF), also appears to play a significant role [3,9,16]. In addition, epigenetic mechanisms, such as DNA methylation, are increasingly recognized for their critical role in shaping the biological underpinnings of psychiatric disorders and, in particular, in anxiety disorders [17]. Other mechanisms, such as histone modifications and non-coding RNAs, facilitate the dynamic interaction between genetic predispositions and environmental influences and shed light on how stress and other environmental factors shape vulnerability to anxiety and related conditions [18,19].

Although significant research has been devoted to uncovering the molecular underpinnings of psychiatric disorders, much remains unclear. For instance, a study that focused on late-life depression (LLD) suggested that serotonin-based treatments may be insufficient for effectively managing LLD, as this disorder is likely associated with additional, distinct biological mechanisms [20]. Additionally, recent investigations into the biological mechanisms underlying CBT have identified epigenetic changes and immune system alterations as potential markers of therapy success [21].

A limited understanding of the underlying brain-based processes has hindered progress in addressing neuropsychiatric disorders and has resulted in the widespread use of similar pharmacological treatments across various mental health conditions, despite many patients experiencing limited efficacy or adverse side effects [22]. To overcome this limitation, ongoing research aims to explore the etiopathogenesis of these disorders and identify reliable biomarkers, aspiring to develop more accurate diagnostic and therapeutic approaches [23,24]. For instance, a genome-wide meta-analysis on anxiety disorders identified 14 risk loci, potential causal variants, and genes associated with anxiety disorders; in particular, it was shown that CTNND1 knockdown affected dendritic spine density and induced anxiety-like behaviors in mice, offering valuable insights into the genetic architecture of anxiety disorders [25].

Τhis review provides a comprehensive overview of the latest—i.e., last decade—literature, both clinical and preclinical, regarding the molecular basis and fundamental aspects of the genetics of anxiety-related traits and anxiety disorders. Taking into consideration the enormous impact of anxiety on human well-being and functionality, the academic importance of this study lies in synthesizing the most recent accumulated knowledge in the field. By illuminating the pathophysiology of this kind of disorders, this review aims to shed further light on the development of innovative therapeutic approaches, ultimately enhancing mental health outcomes and improving the overall quality of life [12,26].

## 2. Materials and Methods

The Preferred Reporting Items for Systematic Reviews and Meta-Analyses (PRISMA) checklist was used to guide this study. The study protocol was registered at the PROSPERO 2025 CRD420251067148. Available from https://www.crd.york.ac.uk/PROSPERO/view/CRD420251067148 (accessed on 26 May 2025).

### 2.1. Search Strategy

Two databases (MEDLINE and Scopus) were selected to carry out the present literature search, which was conducted by two investigators (MP, AS). To trace all relevant studies published between 1 January 2014 and 2 December 2024, the following keywords were used: ((molecular basis) OR (molecular base) OR (neuroendocrine mechanisms) OR (Genetics [MeSH Terms]) OR (epigenetic) OR (epistatic) OR (miRNA)) AND ((anxiety) OR (anxiety disorders) OR (generalized anxiety) OR (obsessive) OR (OCD) OR (GAD) OR (panic)) NOT review [publication type] NOT systematic review [publication type] NOT Meta-Analysis [publication type] on MEDLINE and ((molecular basis) OR (molecular base) OR (neuroendocrine mechanisms) OR (Genetics [MeSH Terms]) OR (epigenetic) OR (epistatic) OR (miRNA)) AND ((anxiety) OR (anxiety disorders) OR (generalized anxiety) OR (obsessive) OR (OCD) OR (GAD) OR (panic)) on Scopus, limited to original articles. All results were limited to English. All retrieved articles were also hand-searched for any further potential eligible articles. Any disagreement regarding the screening or selection process was solved by a third investigator (EM) until a consensus was reached. To minimize the risk of bias, we systematically selected peer-reviewed studies based on predefined inclusion criteria

### 2.2. Selection Criteria

Only full-text original research articles published in the English language were included. Secondary analyses, reviews, guidelines, notes, errata, letters, meeting summaries, comments, unpublished abstracts, and retracted articles were excluded. There was no restriction on study design or other sample characteristics.

### 2.3. Data Extraction

Data extraction was performed independently by four investigators from the team (AB, AS, MP, and IM) using a predefined data form created in Excel.

We recorded the title, the authors, the year of publication, the type of study, demographics, the type of anxiety disorder (if present), the molecular pathway or mechanism, and, finally, the main findings of each study. Possible discrepancies during data extraction were solved via discussion with a third investigator (EM).

### 2.4. Data Analysis

No statistical analysis or meta-analysis was performed due to the high heterogeneity among the studies. Thus, the data were only descriptively analyzed.

## 3. Results

### 3.1. Database Searches

**Figure 1:**
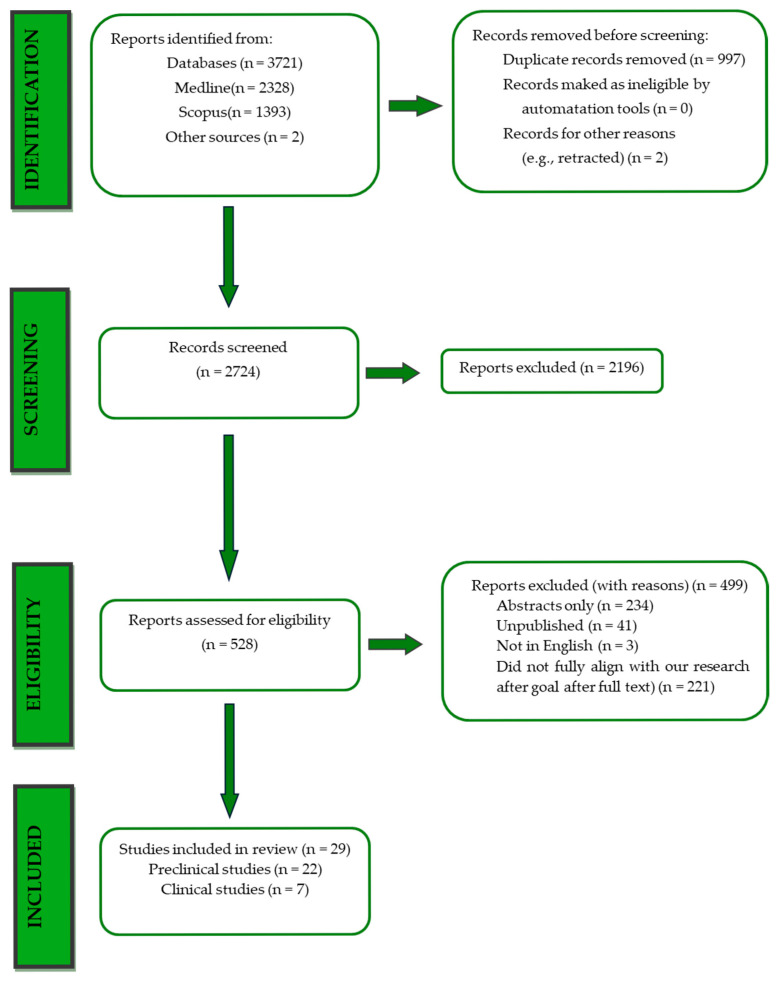
PRISMA flowchart.

Overall, 3723 records were retrieved from the database search. Duplicates were removed; hence, a total of 2756 articles were selected. After dismissing irrelevant studies and screening the full texts of the articles, 29 studies were eligible for inclusion: [27,28,29,30,31,32,33,34,35,36,37,38,39,40,41,42,43,44,45,46,47,48,49,50,51,52,53,54,55] (Figure 1).

### 3.2. Type of Study

Among the publications included, 22 were classified as preclinical (in vivo) trials. Two of the preclinical studies were non-randomized controlled trials (controlled animal studies) and one fell under the category of preclinical case control study. In total, four were randomized controlled trials, one of which was a preclinical RCT. One publication was network pharmacology and molecular docking-based, studying rats and marmosets, and two were observational studies (cross-sectional/retrospective) conducted on both rats (one cross-sectional) and on humans. Three were cohort studies (humans), and one was an in silico study (computational models). Details of types of studies are summarized in Table 1.

### 3.3. Demographics

All preclinical studies were performed on rodent models, specifically mice and rats. Wistar and Sprague-Dawley strains were chosen as rat models, and one study has incorporated marmosets as an additional species. Studies utilizing molecular docking and network pharmacology analyses were all based on rat data.

The remaining studies were conducted on human subjects with diverse demographic characteristics. One study was conducted on adult premenopausal women and men with or without remitted alcohol dependence. Another one was on male patients with a diagnosis of alcohol withdrawal syndrome, whereas yet another was on females in the age group of 18–27 years with a history of abusive childhood. One was conducted on PTSD-diagnosed women.

Two of the studies were conducted on pediatric populations. One of the studies consisted of two cohorts: the first included infants exposed to maternal antidepressants, anxiety, depression, and cord blood DNA methylation, and the second included infants with similar characteristics without anxiety and depression.

### 3.4. Type of Anxiety Symptoms/Anxiety Disorder

Only thirteen studies specified the type of anxiety type and/or symptomatology. Two were focused on post-traumatic stress disorder (PTSD), while one included PTSD, depression, and alcohol dependance. One study investigated anxiety as a symptom of depression, and one as a symptom of alcohol withdrawal. Moreover, one study investigated panic disorders, one studied predatory sound-induced anxiety, and one studied stress associated with the absence of motherly love and the presence of childhood abuse. Finally, one study explored the effects of anxiety during pregnancy, one explored stress-induced anxiety in rats, and one explored anxiety-like behavior related to obesity-induced neuroinflammation.

### 3.5. Molecular Pathway/Mechanism

The most frequent molecular pathways, mechanisms, and genes that appeared in these studies were the GABA signaling pathway (eight preclinical studies); the HPA axis—ACTH and CORT, including the effects of the FKBP5 gene (five preclinical studies, one clinical study, and one in silico study); and the serotonergic system—5-HT transporter, 5-HT2A, 5-HT2C, 5-HT7, and 5-HT1A receptors, and 5-Htr2a-Kmt2a-sensitive gene (seven preclinical studies and one clinical study). Another protein that appeared tobe heavily involved in anxiety production and was mentioned in the included studies is the translocator protein and its ligands (TSPO pathway, PK11195 antagonist, PK11195 blockade, GD-23, and neurosteroids like progesterone and allopregnanolone) (four preclinical studies).

Other less frequent pathways included the oxidative system and oxidoreductases; the glutamatergic pathway; the brain-derived neurotrophic factor (BDNF) through the TLR4-mediated pathway; epigenetic regulation (H3K4 methylation and H3K9 acetylation); the melatonin receptor and neuroinflammatory markers; the nitric oxide (NO) and cAMP pathways; various polymorphisms in CYP enzyme genes; the endogenous cannabinoid system; the Hippo and CREB signaling pathways; the oxytocinergic system and the ZNF575 gene; glucocorticoid receptors; dopamine metabolism regulated by MAO enzymes; immunoregulation; tryptophan metabolism; Ca^2+^/calmodulin-dependent protein kinase II alpha (CamKIIα); hM3Dq DREADD (Designer Receptors Exclusively Activated by Designer Drugs) receptors; and blood–brain barrier impairment due to obesity, influenced by PPAR-α.

### 3.6. Testing Methods

Six studies used molecular docking to assess the results. Molecular docking is a computational technique used to predict the preferred orientation and binding affinity of one molecule when it interacts with another [56]. Pharmacological analysis (pharmacokinetic analysis/inhibition/activation) was another popular method, which was used in seven studies. Other methods include western blot, blood gene expression changes between subjects, cortisol and ACTH levels (blood), Surface Plasmon Resonance, isolation of RNA, Quantitative Real-time PCR, blood-based DNA methylation levels in certain genes, epigenome-wide association studies, chromatin immunoprecipitation, microarray analysis, quantitative reverse transcription PCR, protein interaction analysis, and neuronal deletion of Kmt2a/Mll1 histone methyltransferase in the ventral striatum of mice.

### 3.7. Main Findings

#### 3.7.1. Hypothalamic–Pituitary–Adrenal Axis

The HPA axis has emerged as an untapped pathway for anxiety treatment due to its functions regarding the stress response system. Haj-Mirzaian et al. [27] showed that lithium decreased the activity of the HPA axis in social stressed mice by remodulating the overexpression of IL-1β and reducing NO production through nitric oxide synthase (iNOS) in the prefrontal cortex and hippocampus, primarily by suppressing NOS. This indicates that lithium could potentially modulate neuroinflammatory pathways responsible for stress. Asadi-Pooya et al. [28] investigated FKBP5, a negative regulatory protein of the glucocorticoid receptor (GR) signaling pathway, dysregulation of which has been associated with chronic stress. Through in silico screening of FKBP5 inhibitors for 28 FDA approved drugs, they found that fluticasone propionate, mifepristone, and sertraline possessed adequate FKBP5 binding affinity and were permeable to the blood–brain barrier. It has thus been suggested that inhibiting FKBP5 might enhance the sensitivity of GR and thereby promote HPA regulation and neuroplasticity. Moreover, Chao-Wei Chen et al. [29] showed that ramelteon, a melatonin receptor agonist, reduces the expression level of GR.

On another note, Lama et al. [30] found that palmitoylethanolamide restored the balance of several HPA axis markers, blocked the rise of several peripheral inflammatory mediators, and lowered the activation of microglia and astrocytes, thus preventing the neuroinflammatory cascade in obese mice subjected to a high fat diet. In these mice, both the activity of the HPA axis and the levels of cytokines TNF-α and IL-1β, which were associated with stress pathology, were profoundly elevated. Furthermore, the mechanistic studies of PEA’s action showed that these positive effects were obtained through a PPAR α-dependent pathway, which makes palmitoylethanolamide promising as a novel treatment for HPA axis dysfunction and anxiety, related to obesity. Resveratrol was found to be effective against anxiety when combined with SSRIs, but not when administered alone, which was attributed to the regulation of the HPA axis [40].

Chakraborty et al. [54] sought to determine the impact of N-acetyl cysteine (NAC) on anxiety behavior linked to depression triggered through neonatal clomipramine (CLI) exposure in rats. NAC was shown to be effective in the reduction of plasma corticosterone levels, which are characteristically elevated in states of anxiety in adult rats. Alongside this, chronic NAC treatment led to the reversal of adrenal hypertrophy. Moreover, the effects were comparable to fluoxetine, while both medications significantly reduced anxiety (*p* < 0.001) and decreased corticosterone levels.

K. Roseberry et al.’s [31] study aimed to identify blood biomarkers that can predict treatment response and severity in various anxiety disorders. An important finding was the function of NTRK3 (Neurotrophic Receptor Tyrosine Kinase 3), in the regulation of the HPA axis through neurotrophins like BDNF. Medications that modulate NTRK3 include lithium, omega-3 fatty acids, and antidepressants, which are commonly used for affective disorders and suicidality. Notably, although it was not directly linked to the HPA axis, DYNLL2 was the only one of the top biomarkers that was modulated by benzodiazepines, though in the opposite direction to what is normally observed in high-anxiety conditions. Moreover, estradiol was found to be a potential novel therapeutic tool for anxiety, something that underlines the important role of estrogen receptors (ESR1/ESR2) in anxiety regulation. These significant discoveries potentially open new directions in anxiety treatment, especially using NTRK3-targeting medication and hormonal approaches such as estradiol.

In relation to hormones, Riem et al. [32] found that intranasal oxytocin significantly reduced anxiety and cortisol (*p* < 0.001) levels during recovery in a virtual Trier Social Stress Test (TSST), especially in individuals with higher childhood trauma when paired with social support, suggesting that oxytocin potentially serves as a neurobiological mechanism for facilitating social connection under stress.

Regarding sex differences, Robert M. Anthenelli et al. [33] investigated the differential hormonal responses of men and women in pharmacological challenges used to probe the HPA axis. Results show that women had stronger ACTH and cortisol responses, particularly during the Dex/CRF test, and a slower return to baseline cortisol levels, indicating higher sensitivity to centrally mediated stress pathways, regardless of their dependence. Notably, men showed greater adrenal sensitivity on citalopram tests.

Several studies explored the role of the Translocator Protein (TSPO) in regulating the HPA axis through neurosteroid synthesis. Gudasheva et al. [34] developed GD-23, the first dipeptide ligand for TSPO, which demonstrated anxiolytic effects in animal models. Mice treated with GD-23 spent more time in the center of an open field and explored the open arms of an elevated plus maze more frequently—both indicators of reduced anxiety. This effect was confirmed to be TSPO-dependent, as it was fully blocked by the TSPO antagonist PK11195. Another study by Gudasheva et al. [35] showed that GD-23 crosses the blood–brain barrier (BBB) and has anxiolytic effects comparable to diazepam (*p* < 0.05), likely mediated through TSPO receptor activation and neurosteroid synthesis.

Additional studies explored the role of the Translocator protein (TPSO) in the regulation of the HPA axis through neurosteroid synthesis. Gudasheva et al. demonstrated that GD-23, a dipeptide ligand targeting TSPO, exhibited anxiolytic effects in animal experiments [34]. Mice administered GD-23 displayed reduced anxiety-like behaviors, including increased exploration of the elevated plus maze’s open arms and spending more time in the center of an open field. Gudasheva et al. [35] also showed, in a different study, that GD-23 crossed the BBB and had anxiolytic effects comparable to the ones of diazepam, most likely through TSPO receptor activation and the synthesis of neurosteroids. Likewise, Deeva et al. [36] reported that GD-102, another dipeptide ligand for TSPO, significantly reduced anxiety-like behavior in animals, as indicated by enhanced exploration in both the open field and elevated plus maze (*p* < 0.05).

Mice treated with GD-102 showed increased exploration in both the open field and elevated plus maze tests. Notably, GD-102 had a higher binding affinity to TSPO than previous compounds, and its anxiolytic effects were completely abolished by PK11195, confirming TSPO involvement.

Bojun Xiong et al. [37] examined koumine, an alkaloid from Gelsemium elegans, for its anxiolytic potential in animals exposed to predatory sound stress. Koumine treatment significantly reduced anxiety-like behaviors and lowered plasma ACTH and corticosterone levels; its anxiolytic effects were linked to TSPO interaction.

Lastly, Run Zhao et al. [38] investigated the mechanisms of Qiangzhifang (QZF), a traditional Chinese medicine formula, in treating panic disorder (PD) and identified the 84 active components that target 97 key genes, with top targets including AKT1, FOS, and APP. Key active ingredients, including quercetin and β-sitosterol, showed strong binding affinities to cAMP-PKA signaling pathways. Given that the cAMP-PKA pathway is closely linked to HPA axis regulation, these findings suggest that QZF may exert its anxiolytic effects, in part by modulating stress-related neuroendocrine pathways.

#### 3.7.2. Serotonin

Kitaichi et al. [39] studied DSP-6745, a compound which blocks the serotonin transporter SERT and acts as an antagonist at 5-HT2A, 5-HT2C, and 5-HT7 receptors, thus, increasing the levels of 5-HT, norepinephrine (NE), dopamine (DA), and glutamate in the brain and, more specifically, in the prefrontal cortex. Tests of social interactions showed that the compound reduced stress behaviors shortly after the administration of the drug. Similarly, in another study by Karolina Pytka et al. [55], HBK-14 and HBK-15, two 5-HT1A and 5-HT7 receptor antagonists, were tested by using the Four-Plate Test and elevated plus maze (EPM); the results showed important anxiolytic-like effects.

Additionally, Tseilikman et al. [40] showed that the chronic exposure to stress can increase the levels of serotonin and upregulate the serotonin transporter SERT, as well as the 5-HT3A genes. The authors found that SSRIs alone could not prevent anxiety or decrease serotonin levels, partly because of the suppression of SERT expression. Even though resveratrol upregulated SERT and 5-HT3A expression less than SSRIs, it effectively reduced anxiety and restored serotonin levels, likely through the upregulation of monoamine oxidase A (MAO-A) expression. On the same basis, a different study by Shen et al. [41] based on transcriptomics indicated that the 5-Htr2a receptor of serotonin was downregulated due to anxiety, further supporting the function of serotonin receptors in anxiety regulation. In Run Zhao et al.’s study [38], Qiangzhifang led to an improvement in panic disorders, with a strong binding affinity in the serotonin pathway.

Except for serotonin, Yang et al. [42] studied 2o, a CBD derivative with a double role as an agonist at both CB2 and 5-HT1A receptors. In the stress-induced hypothermia test (SIH), 2o noted significant dose-dependent anxiolytic effects, similar to those of diazepam.

On another note, a study by Chao-Wei et al. [29] that focused on ramelteon, a melatonin receptor agonist (MT1/MT2), showed that it significantly improved anxiety behaviors and social stress in mice in a PTSD model. Notably, ramelteon reduced MAO-A/B levels and increased antioxidant expression in the hippocampus, mechanisms that may contribute to its anti-anxiety effects. Behavioral improvements with ramelteon were highly significant (*p* < 0.0001), suggesting its potential for treating PTSD and anxiety disorders. Further supporting serotonin’s role in anxiety, K. Roseberry et al. [31] identified SLC6A4 (serotonin transporter gene) in their research on blood biomarkers for anxiety and relevant treatment response.

Herbal medicine has also been explored for its serotonin-related anti-anxiety-like effects. Xiacong Xu et al. [52] studied Suanzaoren Decoction (SZRT) and identified 22 key molecular targets. The compound with the strongest binding affinity was MAO-B, linking SZRT to serotonin-related mechanisms, particularly HTR1A (serotonin receptor) and SLC6A4 (serotonin transporter). Their findings also revealed SZRT’s influence on monoamines, amino acids, the MAPK signaling pathway, and inflammatory markers (IL1B, TNF, TP53) (KEGG analysis, *p* < 0.05).

#### 3.7.3. GABA

GABA regulates anxiety based on the inhibitory effect that it has on neurotransmission. Mullally et al. [43] explored the anxiolytic effects of the ethanolic extract of Piper amalago, a traditional Q’eqchi’ Mayan remedy, and found it to act as a GABA_A-BZD receptor agonist by displacing 50% of [3H]-flunitrazepam. In behavioral tests, the compound exhibited similar reduction in anxiety as diazepam. On the same basis, Shantanova et al. [44] found that extracts of *Rhaponticum uniflorum* and *Serratula centauroides* reduce levels of stress hormones (e.g., cortisol, adrenaline), inhibit oxidative stress, and prevent neuronal damage in the cerebral cortex, which is typically triggered by prolonged stress. In a series of behavioral tests, a reduction in anxiety and depressive symptoms, as well as an improvement in exploratory behavior, was noted. 20-hydroxyecdysone, which is abundant in these extracts, showed GABAergic activity, which was also demonstrated by its blockade by bicuculline. Likewise, in Pawar et al.’s [45] study, tetrahydrocarbazoles (THCs), 8a and 8b, were found to have dose-dependent effects on anxiolysis in mice, without corresponding adverse effects. Anxiolytic effects were dose-dependent and were found to be partially mediated by the GABAergic system, as a GABA antagonist, bicuculline, blocked their action. These outcomes demonstrate the efficacy of THCs as lead compounds in the new anxiety treatments with fewer side effects. Similarly, Qiangzhifang alleviated panic disorder symptoms, partially through its binding affinity to the GABA receptor [38].

In terms of genetics, K. Roseberry et al. [31] found that GAD1 (encoding glutamate decarboxylase 1, an enzyme critical for the synthesis of GABA) modestly predicted clinically severe anxiety in all patients in the independent test cohort. GABRA1 was also correlated with the anxiolytic mechanism of Suanzaoren Decoction (SZRT) [52].

#### 3.7.4. Epigenetics

Pape et al. [46] implied that epigenetic changes might help to predict treatment response in PTSD. Specifically, women with higher DNA methylation levels in the CRHR1 gene showed better response to the CRF1 receptor antagonist GSK561679, a drug targeting the body’s response system to stress. Moreover, the methylation levels of the key stress-regulating gene NR3C1 were associated with a significant symptom improvement. All the above underline that DNA methylation can have a useful application as a biomarker, to personalize and improve therapeutical approaches to PTSD treatment. In addition to this, Cardenas et al. [47] analyzed replication between two independent cohorts. They confirmed that the DNA methylation at a CpG site located on the ZNF575 on chromosome 19 had specifically decreased in infants when the mother was prescribed antidepressants while pregnant. This study suggests that these epigenetic findings may have a crucial role in passing anxiety behaviors to the offspring, and they provide a possible source for developing targeted interventions.

In the context of histone modifications, Erica Y. Shen et al. [41] showed that the deletion of a histone methyltransferase (Kmt2a/Mll1) led to a defective spike in medium spiny neurons and induced anxiety behaviors in mice, as was demonstrated in an elevated plus maze, light/dark box, and open-field tests (*p* < 0.05). Specifically, it was linked to reduced histone H3 lysine 4 methylation in key promoter regions. Similarly, Montesinos et al. [48] found that exposing mice to ethanol led to an alteration of acetylation of BDNF and FOSB genes in the prefrontal cortex, reducing anxiety and alcohol preference in adulthood. Moreover, mice with TLR4 were completely protected from the effects, indicating its role in anxiety, at least regarding ethanol use, and indicating the importance of the immune system as a marker for potential therapeutic use in the future.

Finally, in a study on acute ethanol exposure, Tara L. Teppen et al. [49] showed that it greatly decreased miR-494 levels in the amygdala. As a result, chromatin remodeling proteins (CBP, p300, Cited2), particularly molecules associated with the CREB signaling pathway, were upregulated. Furthermore, after using an antagomir to block miR-494, the result mimicked the anxiolytic effects of alcohol in animal models.

#### 3.7.5. Pharmacogenomics

Pharmacogenetics, i.e., the study of how genetics influence drug response, is increasingly being explored to optimize anxiety and depression treatments, minimizing side effects and enhancing efficacy.

Poweleit et al. [50] found that certain genetic variations in HTR2A may lead to better symptom relief from depression and anxiety while also reducing side effects. Specifically, HTR2A rs6313 was significantly associated with maximum sertraline dose and response dose. Moreover, certain CYP2C19 genes were linked to a lower risk of side effects. All in all, findings suggest that pharmacogenetic testing could help to tailor sertraline treatment in pediatric patients, optimizing response and minimizing adverse effects.

Similarly, Michael S. Zastrozhin et al. [51] used a clinical decision support system, including CYP genes, and found that the group that used the system had stronger reductions in anxiety from withdrawal and fewer side effects compared to controls. The authors suggest that poor metabolizers require lower doses to avoid toxicity and rapid ones need higher dosage to become effective. Personalized dosing based on genetics optimizes treatment, improving both efficacy and safety.

Our review’s results and main findings are summarized in Table 2. 

## 4. Discussion

This review examined the molecular basis of anxiety and its implications in the potential development of new treatments. We report research findings from the last decade on the GABAergic pathway and serotonin, HPA axis, epigenetic modifications—such as DNA methylation, histone acetylation, and microRNAs—as well as pharmacogenomics. These results could potentially pave the way for new therapeutic interventions and personalized treatments, according to the patient’s genetic and epigenetic profile.

The role of HPA axis in anxiety regulation appears to be critical in several of our included studies [27,28,29,30,31,32,33,34,35,36,37,38,40,54]. Numerous agents, including lithium, FKBP5 inhibitors, melatonin receptor agonists, and palmitoylethanolamide, have demonstrated good potential in modulating its activity and the relevant neuroinflammatory pathways. Patients with anxiety frequently exhibit elevated cortisol and impaired feedback regulation, consistent with our study findings. Furthermore, commonly used antidepressants seem to indirectly modulate the HPA axis, suggesting that medications that directly affect the axis, such as CRF agonists of FKBP5 inhibitors, could be a promising treatment intervention for anxiety disorders [56].

More specifically, TSPO was the main research goal in four of our studies [34,35,36,37], related to neuroinflammation and HPA axis. A recent review about its utility suggested that it plays a crucial role in the synthesis of neurosteroids, such as allopregnanolone, by facilitating the transport of cholesterol into mitochondria [57], while TSPO ligands have been utilized in positron emission tomography (PET) to assess microglial activation and neuroinflammation in stress-related disorders [57]. In addition, as reported in our included studies, TSPO ligand etifoxine has demonstrated anxiolytic effects in animal models, influencing emotionality, stress reactivity, and neurosteroid level. Thus, compounds such as etifoxine and GD-23 that target TSPO show potential as novel non-benzodiazepine treatments for anxiety [34,35,58], given their role in neurosteroid synthesis and the modulation of neuroinflammation.

Several studies supported the role of the glucocorticoid receptor (GR) in anxiety [28,29,46]. This finding is in line with a systematic review suggesting that increased methylation of NR3C1, the gene that encodes GR, is associated with decreased GR expression, which may contribute to anxiety pathophysiology, particularly in individuals exposed to early-life stress [59].

On a genetic level, our included studies have identified certain genes related to anxiety (e.g., BDNF, GABRA1, GAD1, SLC6A4, 5-HT3A, AKT1, FOS, and APP); these are in concordance with a recent systematic review suggesting that BDNF, PERIOD2, and SLC6A4 were correlated, not only with anxiety, but with depression and addictions as well [60]. An analysis of the interactions among the 51 genes further highlighted BDNF and SLC6A4 as key contributors to the development of anxiety, depression, and addiction [31,38,40,48,60]. These findings emphasize the interconnected genetic basis of these disorders, suggesting that targeting BDNF and SLC6A4 could offer broad therapeutic benefits with a potential for integrated treatment approaches, combining pharmacological, genetic, and behavioral interventions to address overlapping neurobiological mechanisms. At the same time, the above findings emphasize the inherent heterogeneity and the translational gaps of research into the molecular basis of anxiety, where species-specific differences in neurocircuitry, genetic regulation, and the behavioral expression of anxiety can pose significant challenges in moving findings from preclinical models to effective human treatments.

On a neurotransmitter/receptor level, two of our studies supported the anxiolytic implications of the 5-HT7 receptor antagonists [39,55]; this is also supported by a systematic review, where anxiolytic effects of 5-HT7 antagonist agents were noted in animal models [61]. In addition, two studies highlighted the role of MAO in anxiety. Tseilikman et al. [40] suggested that elevated serotonin levels, along with an increased expression of serotonin transporter and 5-HT3A receptors, contribute to stress-related anxiety. However, co-treatment with resveratrol and the antidepressant-selective serotonin reuptake inhibitor sertraline had the strongest anxiolytic effect, likely due to MAO-A upregulation, which helped to restore serotonin balance. In contrast, Chao-Wei Chen et al. [29] implied that ramelteon reduced MAO-A/B levels and increased antioxidant expression in the hippocampus, suggesting a different pathway for its anti-anxiety effects.

Simultaneously targeting CB_2_R and 5-HT_1_AR with a single agent has emerged as a potentially effective approach for treating anxiety-related conditions, potentially offering enhanced efficacy through the simultaneous modulation of two relevant pathways [42]. CBD has shown therapeutic potential in a wide range of psychiatric symptoms, including anxiety, which has been partly attributed to its interaction with the 5-HT1A receptor; this appears to happen in a dose-dependent fashion, suggesting that different dosages of CBD may have varying therapeutic outcomes [62].These findings emphasize the complexity of anxiety regulation and the potential for multi-target therapeutic strategies. Overall, the modulation of serotonergic receptors, MAO activity, and cannabinoid interactions suggests that combining different mechanisms may enhance anxiolytic efficacy.

Regarding GABAergic activity, in six of our studies, preparations were used to reduce anxiety via a GABAergic mechanism [31,38,43,44,45,52]. Our findings indicate that GABA-A receptors are particularly important in anxiety regulation [43,44], which is consistent with the existing literature [63]. Neurosteroids such as allopregnanolone, which is controlled by TSPO [58], have the ability to alter GABA-A receptors and GABAergic signaling and thus have anxiolytic effects. As such, the future investigation of TSPO-targeted neurosteroids and other GABA receptor modulators as potential treatment options for anxiety disorders is warranted, since novel pharmaceutical agents that target the GABA system may offer more effective outcomes with fewer side effects [63].

Four of our studies have used traditional medicine plants to treat anxiety, with encouraging results in the molecular pathways known to be associated with anxiety [38,43,44,52]. Our findings suggest that phytomedicines can act through pathways beyond GABAergic transmission, such as serotonergic signaling, the PI3K-Akt [46], and the cAMP-PKA signaling pathway [41]. Mullally et al.’s study indicates that they could also influence the HPA axis [47], whilst they appear capable of regulating monoaminergic systems and exerting antioxidant activity [48]. Although preclinical research has highlighted these diverse mechanisms, including modulation GABA receptors, further carefully designed human clinical trials are needed to determine their therapeutic potential [64]. Medicinal herbs such as Silexan may be a useful adjunct therapy for anxiety disorders; however, additional research is required to validate these findings and establish consistent dosing protocols [65].

On another note, a number of our studies supported the role of epigenetics in regulating anxiety [41,47,48,49]. Stressful experiences have been shown to alter DNA methylation, especially in genes that govern HPA axis function and stress reactivity [66]. These alterations can influence behavior and cognition, potentially increasing susceptibility to anxiety disorders. Epigenetic modifications, including DNA methylation and histone modifications, can affect the expression of BDNF and other genes, thereby influencing the anxiety phenotype [18,66]. Moreover, preliminary data suggest that HDAC inhibitors have demonstrated potential therapeutic value for anxiety disorders, as observed in both animal models and early-stage human trials [67].

In the context of epigenetics, miR-494 is mentioned in one of our included studies as a central modulator of ethanol-induced anxiolysis and a potential therapeutic target for anxiety and alcohol use disorders [49]. Recent research suggests that the amygdala, hippocampus, and prefrontal cortex regions of the brain associated with anxiety have dysregulated expression of certain miRNAs [68,69]. In addition, these miRNAs influence the expression of genes related to neurotransmitter systems, stress responses, and synaptic plasticity. In animal research, some specific miRNAs (for instance, miR-34, miR-144, and miR-132) have also been associated with anxiety-like behavior [68]. Epigenetic mechanisms play a crucial role in shaping anxiety susceptibility by regulating key genes involved in stress responses, neurotransmission, and neuroplasticity. Targeting epigenetic pathways, including histone deacetylase inhibitors (HDACi) and miRNA modulators, presents a promising avenue for developing novel, mechanism-based anxiety treatments.

Finally, regarding pharmacogenomics, two of our included studies demonstrated how variations in HTR2A and CYP2C19 genes influence sertraline efficacy and tolerability in bromdihydrochlorphenylbenzodiazepine dosing in pediatric and in adult populations [50,51]. This is in line with existing evidence, emphasizing that genetic markers in serotonin receptor and CYP450 pathways are predictive of antidepressant response [70]. On the same basis, a genome-wide association study (GWAS) identified various genetic variants, such as the serotonin transporter, BDNF, and interleukin-11 gene, associated with differential treatment responses, further highlighting the potential of genetic markers in the treatment of anxiety disorders [71].

Our review has a number of limitations. Rendering preclinical studies to human patients is difficult due to neurobiological differences and reliance on animal models that do not fully account for the heterogeneity of anxiety. The studies analyzed were highly diverse, covering the spectrum of neurotransmitter signaling, neurosteroids, inflammatory mediators, and epigenetic modifiers, making direct comparison and universal treatment targets difficult, while methodologies also vary, particularly in phytomedicine and novel drug research. Although the potential of multi-target therapies exists, the interactions between the different pathways have not yet been sufficiently explored. The future of personalized treatment lies in genetic and epigenetic markers, but these would need larger-scale trials to be confirmed.

## 5. Conclusions and Future Directions

In conclusion, preclinical and clinical evidence from the last decade indicates that the molecular basis of anxiety is driven by intricate interactions among neurobiological mechanisms, genetic predispositions, and environmental modifiers. GABAergic and serotonin mechanisms remain main modulators and subsequently key treatment targets for anxiety. More novel approaches, such as the dual targeting of cannabinoid and serotonin receptors, offering new possibilities, have also been brought to light. The HPA axis has been shown to have a critical role in anxiety, according to interventions that target glucococticoid signaling and modulate neuroinflammation. Epigenetic mechanisms have also been highlighted, suggesting that they might contribute to anxiety-related phenotypes and could also be incorporated in therapeutic approaches. On the same note, pharmacogenomics, regarding serotonin receptors and CYP450 genes, further emphasize the need for personalized medicine.

Future research should prioritize developing drugs that target the HPA axis to restore stress response balance and investigating epigenetic interventions to advance personalized treatments. In addition, new human studies beyond preclinical trials in mice and randomized controlled trials in different populations are needed to validate our findings. Further, while traditional GABA-modulating phytomedicines show promise, the lack of well-controlled clinical trials limits their therapeutic validation, requiring further research to assess dosing and long-term efficacy. Finally, the combination of biological treatments such as SSRIs with newer agents that could improve treatment response should also be further explored.

## Figures and Tables

**Table 1:** Type of studies.

Types of Preclinical Studies	22	Types of Clinical Studies	7
Non-randomized controlled trials	2	Randomized controlled trials	3
Case–control study	1	Cross-sectional study	1
Randomized controlled trials	1	Cohort studies	3
Cross-sectional study	1		
In silico study	1		
Network pharmacology and molecular docking	1		
Unspecified	15		

**Table 2:** Summary of the main findings.

Authors/Year	Type of Study	Demographics	Anxiety Type	Molecular Pathway/Mechanism	Testing Methods	Main Findings
A Haj-Mirzaian, S Amiri et al., 2016 [27]	Preclinical experimental study (in vivo)	Male NMRI (Naval Medical Research Institute) mice, weighing 10–14 g and on postnatal day (PND) 21–25	-	Neuroinflammatory and Nitric Oxide (NO) Pathway, HPA axis (cytokine signaling and inducible iNOS activity)		Lithium has a protective role against depressant and anxiogenic effects of juvenile SIS (social isolation stress)
A. Asadi-Pooya, Mahdi Malekpour, et al., 2023 [28]	In silico study	-	Functional seizures (FS)	HPA axis (FKBP51 Protein)	Μolecular docking and pharmacokinetic profiling to identify potential FKBP5 inhibitors	Fluticasone propionate was identified as a good candidate to deliver FKBP5 blockade. Prednisolone and Dexamethasone also have acceptable pharmacological profiles to be used as FKBP5 inhibitors. Some psychiatric drugs (e.g., Mirtazapine, Sertraline, Fluoxetine, and Citalopram) also have acceptable pharmacological profiles to be used as FKBP5 inhibitors
Chao-Wei Chen, Wei-Lan Yeh, et al., 2024 [29]	Preclinical experimental study (in vivo)	C57BL/6 mice (male, 6 weeks old) and male ICR mice (age 20–25 weeks)	Posttraumatic stress disorder (PTSD)	Melatonin receptor activation (expression of monoamine oxidases, glucocorticoid receptors, and endogenous antioxidants in the hippocampus)		The activation of both melatonin and ramelteon regulates RSDS-induced anxiety-like behaviors and PTSD symptoms
Adriano Lama, Claudio Pirozzi, et al., 2023 [30]	Preclinical experiment	Male C57Bl/6J mice. Three groups were studied: control (standard diet), HFD-fed mice, and HFD-fed mice treated with PEA	Anxiety-like behavior related to obesity-induced neuroinflammation	HPA axis (CRH and CRHR1), increased neuroinflamatory markers, impaired blood–brain barrier, altered dopamine and GABA levels; PEA works through PPAR-a activation	Western blot, open-field test	PEA treatment alleviated anxiety symptoms by reducing neuroinflammation, restoring BBB integrity, and rebalancing neurotransmitter levels. Its effects were mediated through PPAR-α activation, making it a promising therapeutic option for obesity-related anxiety
Suwarna Chakraborty, Sunil Jamuna Tripathi, et al., 2020 [54]	Preclinical experimental study	Rats	-	Oxidative stress, glutamatergic, and neurotrophic pathways, mitochondrial and astroglial functioning. HPA axis		It was found that NAC might be a candidate and/or add-on therapy for treating comorbid anxiety, amygdalar hyperactivity, and HPA axis dysfunctions in depression and associated disorders
K. Roseberry, H. Le-Niculescu, et al., 2023 [31]	Cohort of psychiatric subjects	Humans (major psychiatric disorders with changes in state anxiety), (major psychiatric disorders with clinically severe anxiety), (an independent major psychiatric disorders cohort for predicting state anxiety), (future hospitalization with anxiety as the primary reason)		Hippo signaling pathway and CREB signaling pathway	Blood gene expression changes between self-reported low and high anxiety states in individuals with psychiatric disorders	The biomarkers with the best overall evidence were GAD1, NTRK3, ADRA2A, FZD10, GRK4, and SLC6A4. They identified which of their biomarkers are targets of existing drugs, and thus can be used to match patients to medications. They also used their biomarker gene expression signature to identify drugs that could be repurposed for treating anxiety, such as estradiol
M.M.E. Riem, L.E. Kunst, et al., 2020 [32]	Randomized controlled trial	180 females with abusive childhood from 18 to 27 years old	Stress after motherly love withdrawal and experiences of child abuse	Oxytocinergic system	Trier Social Stress Test (TSST)	Intranasal oxytocin enhances the stress-protective effect of social support during psychosocial stress. The findings point to oxytocin as a neurobiological means to attain social support under stressful circumstances, particularly in women with negative childhood experiences
Robert M. Anthenelli a, Jaimee L. Heffner, et al., 2018 [33]	Randomized controlled trial	Adult premenopausal women and men with and without remitted AD	Alcohol dependence (AD), post-traumatic stress disorder (PTSD), and major depression	Central and peripheral mechanisms regulating sexually-diergic ACTH and cortisol responses via the limbic–HPA axis	Cortisol levels, molecular docking, and pharmacokinetic profiling to identify potential FKBP5 inhibitors	Women and men exhibit diametrically opposite reactions to pharmacological challenges probing 5-HTergic and peripheral mediators of the endocrine stress response, with women mounting greater reactions to Dex/CRF than men and to their own responses to a citalopram stimulation test
T.A. Gudasheva, O. A. Deeva, et al., 2015 [34]	Preclinical experimental study (in vivo)	BALB/cAnN mice and outbred CD1 mice	-	GABA pathway and TSPO pathway (PK11195 blockade)		The results suggest that GD-23 is a ligand of the translocator protein, and its structure can become the basis for creating anxiolytics with a fundamentally new mechanism of action. The stereoselectivity of the anxiolytic effect of GD-23 is demonstrated
T. A. Gudasheva, O. A. Deeva, et al., 2019 [35]	Preclinical experimental study (in vivo)	adult male CD-1 mice BALB/c mice	-	TSPO pathway (GD-23), GABA pathway	Molecular docking of compound GD-23 in the active site of the TSPO receptor using Glide software	GD-23 and its analogues exhibit pronounced anxiolytic and nootropic activities via TSPO-dependent pathways
O. A. Deeva, A. S. Pantileev, et al., 2019 [36]	Preclinical experimental study (in vivo)	BALB/c mice and ICR mice	-	TSPO pathway (PK11195 antagonist)	Pharmacological inhibition, molecular docking	A new dipeptide, N-phenylpropionyl-L-tryptophanyl-L-leucine amide (GD-102), exhibited anxiolytic activity
Bojun Xiong, Zhifeng Zhong, et al., 2022 [37]	Preclinical experiment (in vivo)	Specific-pathogen-free male Wistar rats at 6–8 weeks of age and weighing 140–160 g	Predatory sound anxiety	TSPO-neurosteroids HPA axis (ACTH, CORT)	Surface plasmon resonance (SPR) technology to assess koumine-TSPO affinity, plasma ACTH and CORT levels	Koumine has obvious anxiolytic effect in the PS-induced anxiety model. Targeting TSPO–neurosteroids–HPA axis may be an important mechanism by which koumine exerts its anxiolytic effect
Run Zhao, Pulin Liu, Anran Song, et al., 2021 [38]	Network pharmacology and molecular docking study	Rats	Panic disorder	Serotonergic, GABA, and cAMP signaling pathways		The 5-HT, GABA, and cAMP signaling pathways are important routes by which QZF treats PD, meaning that QZF might have the characteristics of multicomponent, multitarget, and multipathway synergistic effects in the treatment of PD
Maiko Kitaichi, Taro Kato, et al., 2024 [39]	Preclinical experimental study (in vivo and in vitro)	Rats and marmosets	Anxiety as a comorbid symptom of depression	Serotonergic and monoaminergic neurotransmission pathway	In vivo: Forced swim test, social interaction test, spontaneous locomotion, rat apomorphine-induced PPI, marmoset ORD task; in vitro: Measures DSP-6745 binding affinity to serotonin transporter and receptors	DSP-6745 is a multimodal 5-HT receptor antagonist and a 5-HT transporter inhibitor that has the potential to be a rapid acting antidepressant with efficacies in mitigating the comorbid symptoms of depression such as anxiety, psychosis, and cognitive dysfunction
Vadim E. Tseilikman, Olga B. Tseilikman, et al., 2024 [40]	Preclinical experiment	Rats	Stress-induced anxiety	Serotonergic system (SERT, 5-HT3A), MAO-A, BDNF	Εlevated plus maze test, RNA isolation, quantitative real-time PCR	Both heightened serotonin levels and increased SERT and 5-HT3A receptor gene expression play an essential role in stress-induced anxiety. SSRIs proved ineffective in downregulating serotonin levels, yet resveratrol was seen to effectively regulate the pathways. Of great relevance is that treatment with a combination of resveratrol with sertraline produced the strongest anxiolytic response, which reflects their potential therapy for anxiety disorders
Erica Y Shen, Yan Jiang, et al., 2016 [41]	Preclinical experiment and non-randomized control trial	Mice carrying a previously described Mll1flox/floxallele and a CamKII*α*-Cre (CamK-Cre) transgenic line		Serotonergic pathway, H3K4-promoter hypomethylation	Νeuronal deletion of Kmt2a/Mll1 histone methyltransferase in the ventral striatum of mice	*Kmt2a* conditional deletion in postnatal forebrain is associated with absent or blunted responses to stimulant and dopaminergic agonist drugs. *Kmt2a* regulates synaptic plasticity in striatal neurons and provides an epigenetic drug target for anxiety and dopamine-mediated behaviors
Wenjiao Yang, Xudong Gong, et al., 2024 [42]	Preclinical experiment (in vivo)	Male ICR mice	-	Endogenous cannabinoid system, serotonergic pathway	In vitro receptor binding assays, molecular docking, and in vivo behavioral studies in animal models	As a potential therapeutic target for psychiatric disorders, CB2R lacks psychoactive side effects when activated, making it a promising target for the treatment of psychiatric disorders such as depression and anxiety. Their findings highlight the unique dual agonistic activity of compound 2o on CB2R and 5-HT1AR, as well as its favorable oral PK properties
M. Mullally a, C. Cayer, et al., 2016 [43]	Preclinical experiment and case–control trial	Rats		GABAA–BZD receptor ligand with high affinity for the GABA pathway		The ethnobotanical use of this plant may have a pharmacological basis in its anxiolytic activity. *P. amalago* lowered anxiety-like behavior in rats significantly in all three behavioral tests, although not at all doses. For example, the fear extinction process seen in the CER test was effective only at the highest dose
Larisa N. Shantanova, Daniil N. Olennikov, et al., 2021 [44]	Preclinical experiment and non-randomized controlled trial	Mice		GABA pathway, ⭣sympathoadrenal system and HPA-axis, inhibition of free radical oxidation, and enhancement of endogenous antioxidant activity		The plants increase non-specific resistance to emotional stress induced by the single and long-term action of stress factors. In acute emotional stress, the development of Selye’s triad is inhibited, while, in chronic stress, the marked manifestations of anxiety-depressive syndrome were decreased
Tushar Janardan Pawar, Edson E. Maqueda-Cabrera, et al., 2020 [45]	Preclinical experiment (in vivo)	Balb/c mice		GABA pathway		These compounds showed activity against anxiety and mood disorders that can possibly contribute to the discovery of new drugs. In addition, the use of *N*-protected 2-methylindole acrylaldehyde will set a new base for the synthesis of medically and pharmacologically important tetrahydrocarbazoles
Julius C. Pape, Tania Carrillo-Roa, et al., 2018 [46]	Cohort study	57 PTSD-diagnosed women	Post-traumatic stress disorder (PTSD)	*NR3C1* and *FKBP5* (PTSD relevant genes)	Blood-based DNA methylation levels in CRHR1 and NR3C1 genes	The results support a possible role of *CRHR1* methylation levels as an epigenetic marker to track response to CRF1 antagonist treatment. Moreover, pre-treatment *NR3C1* methylation levels may serve as a potential marker to predict PTSD treatment outcome
Andres Cardenas, Sabrina Faleschini, et al., 2019 [47]	Cohort study	Project Viva: 479 infants with history of maternal antidepressant use, anxiety, depression, and cord blood DNA methylation, 580 kids. Generation study: 999 infants with history of maternal antidepressant use and cord blood DNA methylation	Investigates pregnancy anxiety	3 (Zinc Finger Protein 575) was identified as significantly associated with prenatal antidepressant exposure. ZNF575--> role in transcriptional regulation	Εpigenome-wide association studies (EWAS) to analyze DNA methylation in umbilical cord blood and whole blood samples	Maternal antidepressant use was associated with DNA methylation differences at 130 CpG sites, with ZNF575 cg22159528 showing the strongest association. The study did not find consistent DNA methylation associations with maternal anxiety or depression across both cohorts
Jorge Montesinos, María Pascual, et al., 2016 [48]	Preclinical experimental study (in vivo)	Female C57BL/6 WT and TLR4 knockout (KO) mice aged 30 days	-	TLR4-mediated neuroimmune pathway	Molecular analyses included RT-qPCR for gene expression, Western blot for protein levels, and chromatin immunoprecipitation (ChIP) for epigenetic modifications	Role of the TLR4 pathway in the epigenetic changes, along with the long-term anxiety and rewarding effects induced by intermittent ethanol administration in the adolescence
Tara L. Teppen, Harish R. Krishnan, et al., 2016 [49]	Preclinical experiment and randomized controlled trial	Adult male Sprague-Dawley rats used in this study were purchased from Harlan Laboratories		miR-494⭣ after ethanol-induced anxiolysis; CBP, p300, Cited2↑ in amygdala; antagomir: Cited2, CBP, p300, H3K9ac↑, mimicking anxiolysis; CREB signaling, epigenetic mechanisms	Microarray analysis to profile miRNA expression in the amygdala, quantitative reverse transcription PCR (qRT-PCR), Surface Plasmon Resonance (SPR) technology	Acute ethanol-induced reduction in miR-494 expression in the amygdala can serve as a key regulatory mechanism for chromatin remodeling, possibly leading to anxiolysis
Ethan A. Poweleit, Stacey L. Aldrich, et al., [50]	Retrospective observational study	Pediatric cohort with 352 patients under 19 years of age, 70% female, 30% male	-	Serotonergic system, transporter levels, and glutamatergic transmission	Pharmacogenetic analysis, including blood sampling to evaluate genetic variants and their correlation with the tolerability and response to sertraline	Both pharmacokinetic and pharmacodynamic factors, in addition to clinical and demographic components, influence sertraline dose, response, and tolerability
Zastrozhin MS et al., 2018 [51]	Randomized controlled trial	51 male patients with alcohol withdrawal syndrome	Anxiety symptoms following alcohol withdrawal	Polymorphisms in the genes of CYP enzymes	Pharmacogenomic analysis and personalized clinical decision support system for dosage optimization	It was shown that pharmacogenetic-guided personalization of the drug dose can reduce the risk of undesirable side effects and pharmacoresistance. It allows for recommending the use of pharmacogenomic CDSSs for optimizing drug dosage
Karolina Pytka, Anna Partyka, et al., 2015 [55]	Preclinical experimental pharmacological experiment	Adult male CD-1 mice (18–21 g) and male Wistar rats (170–220 g)	-	Serotonergic system, adrenergic a1 receptors, serotonin transporter		HBK-14 and HBK-15 are promising novel dual 5-HT1A/5-HT7 receptor antagonists with antidepressant- and anxiolytic-like effects. HBK-14 showed greater anxiolytic-like activity than HBK-1. Effects are serotonin-dependent, supporting 5-HT1A receptor blockade as a potential antidepressant/anxiolytic strategy
Xiaocong Xu, Bingbing Gao, et al., 2021 [52]	Cross-sectional study	Rats	-	GABA pathway, oxidoreductases, glucocorticoid receptors, DA, MAO enzymes, immunoregulation, dopaminergic, serotonergic, tryptophan metabolism, and addiction (cocaine, morphine)	Pharmacological database analysis, protein interaction analysis (PPI), molecular docking of active compounds with MAOB	SZRT produces anti-anxiety effects mainly by affecting the neurotransmitter release, transmission, and immunoregulation.
Sthitapranjya Pati, Ankit Sood, et al., 2017 [53]	Preclinical and behavioral experiment	Adult male Sprague-Dawley rats (4–5 months old)	-	CamKIIα-positive excitatory neurons in the medial prefrontal cortex, hM3Dq DREADD receptors, neuronal activation marker c-Fos	Pharmacogenetic activation of Ca^2^⁺/calmodulin-dependent protein kinase alpha (CamKII alpha)-positive excitatory neurons on anxiety-like behavior	Acute activation of excitatory neurons in the mPFC decreases anxiety-like behavior; mPFC excitatory activity modulates downstream circuits implicated in anxiety regulation

## Data Availability

Data are contained within the article.
